# Dysregulated fibrinolysis and plasmin activation promote the pathogenesis of osteoarthritis

**DOI:** 10.1172/jci.insight.173603

**Published:** 2024-03-19

**Authors:** Qian Wang, Guoqiang Shao, Xiaoyi Zhao, Heidi H. Wong, Kate Chin, Mackenzie Zhao, Audrey Bai, Michelle S. Bloom, Zelda Z. Love, Constance R. Chu, Zhen Cheng, William H. Robinson

**Affiliations:** 1 Division of Immunology & Rheumatology, Stanford School of Medicine, Stanford, California, USA.; 2 Veterans Affairs Palo Alto Health Care System, Palo Alto, California, USA.; 3 Molecular Imaging Program at Stanford, Canary Center at Stanford for Cancer Early Detection,; 4 Department of Radiology, Stanford Bio-X Program, and; 5 Department of Orthopaedic Surgery, Stanford School of Medicine, Stanford, California, USA.

**Keywords:** Inflammation, Osteoarthritis

## Abstract

Joint injury is associated with risk for development of osteoarthritis (OA). Increasing evidence suggests that activation of fibrinolysis is involved in OA pathogenesis. However, the role of the fibrinolytic pathway is not well understood. Here, we showed that the fibrinolytic pathway, which includes plasminogen/plasmin, tissue plasminogen activator, urokinase plasminogen activator (uPA), and the uPA receptor (uPAR), was dysregulated in human OA joints. Pharmacological inhibition of plasmin attenuated OA progression after a destabilization of the medial meniscus in a mouse model whereas genetic deficiency of plasmin activator inhibitor, or injection of plasmin, exacerbated OA. We detected increased uptake of uPA/uPAR in mouse OA joints by microPET/CT imaging. In vitro studies identified that plasmin promotes OA development through multiple mechanisms, including the degradation of lubricin and cartilage proteoglycans and induction of inflammatory and degradative mediators. We showed that uPA and uPAR produced inflammatory and degradative mediators by activating the PI3K, 3′-phosphoinositide-dependent kinase-1, AKT, and ERK signaling cascades and activated matrix metalloproteinases to degrade proteoglycan. Together, we demonstrated that fibrinolysis contributes to the development of OA through multiple mechanisms and suggested that therapeutic targeting of the fibrinolysis pathway can prevent or slow development of OA.

## Abstract 2

The fibrinolysis pathway, including urokinase plasminogen activator and its receptor, produces inflammatory and degrative mediators in the development of osteoarthritis.

## Graphical abstract

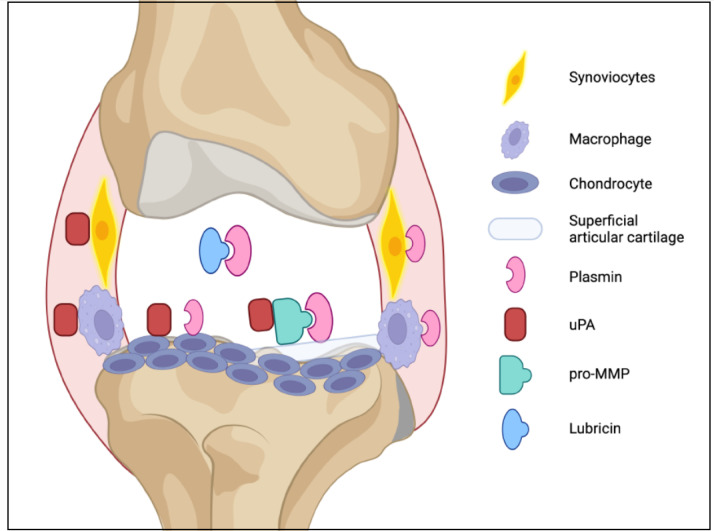

## Introduction

Development of osteoarthritis (OA) is often associated with a preceding joint injury (1–5). OA is the most common joint disorder and a leading cause of disability. OA is characterized by progressive cartilage breakdown, bone remodeling, osteophyte formation, and synovitis (6). The mechanisms by which joint injury predisposes an individual to OA remain elusive. Symptom-modifying therapy and joint replacement are currently the only treatment available for OA (7). There remains a substantial need for safe and effective disease-modifying drugs to prevent or treat OA following joint injury.

Traumatic injury that causes bleeding in the joint (hemarthrosis) triggers activation of fibrinolysis (8). Removal of fibrin is an essential step in wound healing and restoration of tissue function following mechanical or infectious insult (9). Fibrin deposition is reported in OA and is associated with cartilage degeneration via upregulation of a disintegrin and metalloproteinase with thrombospondin motifs 5 (Adamts5) and MMP-13 in chondrocytes (10). During the fibrinolytic process, plasmin, the key serine protease of the fibrinolysis cascade, is generated from zymogen plasminogen, either by the tissue plasminogen activator (tPA) or by the urokinase plasminogen activator (uPA). Active plasmin degrades insoluble fibrin clots and thrombi produced during coagulation, a process known as hemostasis (11). The fibrinolytic system, along with coagulation, is tightly regulated by multiple inhibitors, cofactors, and receptors to ensure balanced hemostasis. For example, circulating plasmin is neutralized by α2-macroglobulin (12) and antiplasmin (13, 14) whereas tPA is blocked from activation by serine protease inhibitors (serpins), such as plasminogen activator inhibitor-1 (PAI-1), and nonserpin inhibitors (15).

The serine protease uPA, and its receptor uPAR, a glycosylphosphatidylinositol-anchored protein, are known to have pleiotropic functions in regulating fibrinolysis, inflammation, and immune responses (16). uPAR is expressed in multiple cell types, including endothelial cells and hematopoietic cells, and is upregulated in response to inflammatory cytokines. uPA is generated as zymogen pro-uPA, which consists of 2 critical domains: i) the amino terminal fragment (ATF), and ii) the catalytic C-terminal domain (16). uPAR binds with high affinity to uPA and pro-uPA through the ATF region, which localizes its proteolytic activity to the cell surface (17). Additionally, uPAR can bind to the extracellular matrix (ECM) protein vitronectin (16), partners with integrin family members (18), and associates with the G protein–coupled receptor, FPRL1 (19), to modulate cell adhesion and migration (20). Dysregulated expression of uPA and uPAR have been implicated in HIV infection (21) and cancer (16, 22–24).

Growing evidence indicates that activation of fibrinolysis plays a major role in OA pathogenesis. Of note, fibrinolysis activation is dysregulated in the later stages of OA (25, 26). Levels of uPA and tPA are higher in OA synovial fluid when compared with healthy synovial fluid (25), and fibrinolytic activity is higher in OA synovial membranes than in normal synovial membranes (26, 27). Furthermore, elevated levels of plasmin activity have been detected in the cartilage of patients with OA, which are normally not present in healthy individuals (28, 29).

In this report, we present evidence that the fibrinolytic pathway, which involves plasmin, tPA, uPA, and uPAR, is dysregulated in OA. Here, we demonstrated that genetic deficiency or pharmacological blockade of plasmin, PAI-1, tPA, uPA, and uPAR altered the progression of OA following destabilization of the medial meniscus (DMM) in mice. Our in vitro studies of human OA identified numerous mechanisms by which plasmin promotes OA development, which includes degradation of lubricin and degradation of cartilage proteoglycans. We also showed that plasmin-activated pro-MMPs accelerated degradation of proteoglycans and induced inflammatory and degradative enzymes in human OA primary synoviocytes. Our results were verified in a DMM mouse model of OA. Similar to plasmin, genetic deficiency of uPA and uPAR attenuated OA-related pathology in DMM mice. Consistent with this finding, microPET/CT imaging detected increased uptake of a uPAR binding peptide, AE105, in mouse OA joints following DMM. Finally, in vitro analyses identified uPA and uPAR as contributors to OA, not only by activating the PI3K, 3′-phosphoinositide-dependent kinase-1 (PDK1), AKT, and ERK signaling cascades, which subsequently induce the production of inflammatory and degradative enzymes, but also by directly degrading proteoglycans and by activating MMPs to degrade proteoglycans in the cartilage. Together, these results demonstrate that fibrinolysis plays a role in the progression of OA. Moreover, it suggests that the plasminogen activation pathway can be targeted by therapeutic approaches to prevent or slow the development of OA following traumatic joint injury.

## Results

### Key molecules in the fibrinolysis pathway are dysregulated in human OA joints.

**Figure 1:**
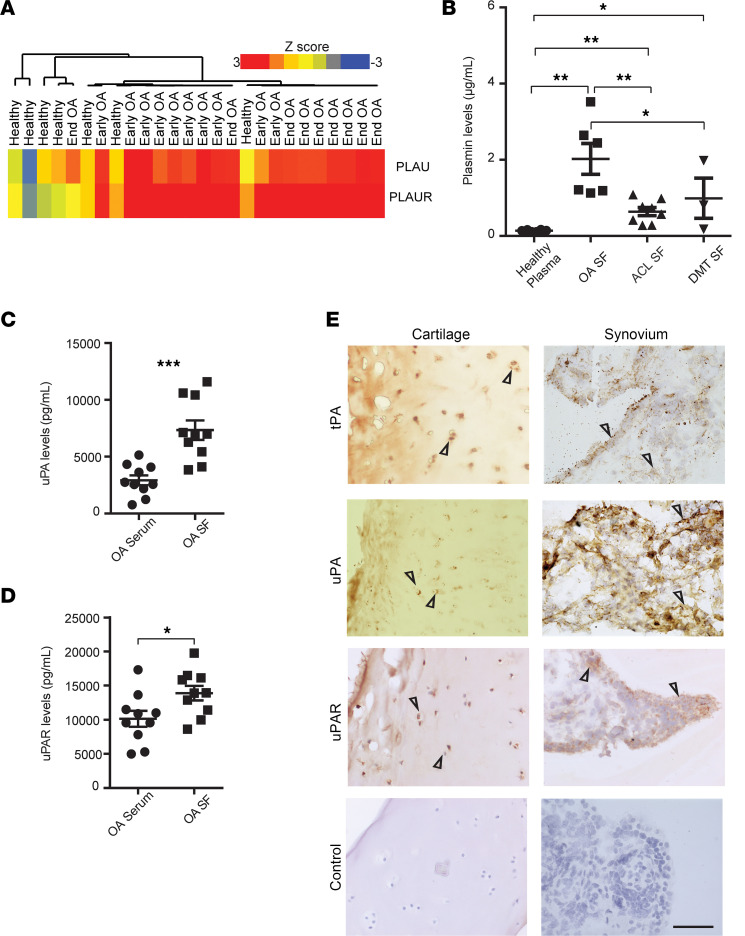
Key molecules in the fibrinolysis pathway are dysregulated in human OA joints. (**A**) Unsupervised hierarchical clustering of uPA and uPAR expression in microarray data set on synovial membranes from healthy individuals (*n* = 7) or those with early- (*n* = 10) or end-stage OA (*n* = 9). Scale bar indicates *z* score. (**B**) ELISA analysis of plasmin levels in knee joint synovial fluids from individuals with OA (*n* = 6), ACL tear (*n* = 8), or DMT (*n* = 3) and in the plasma from healthy individuals (*n* = 8). (**C** and **D**) ELISA analysis of activated uPA (**C**) or uPAR (**D**) levels in synovial fluid of knee joints and serum from individuals with confirmed OA (*n* = 10). (**E**) Representative images from immunohistochemical staining of tPA, uPA, uPAR, and isotype control in damaged knee cartilage (left) and synovium (right) of OA from individuals who underwent total knee replacement. The arrowhead indicates positive staining for tPA, uPA, and uPAR. For panels **B**–**D**, data are the mean ± SEM of duplicates or triplicates and are representative of at least 2 independent experiments. *P < 0.05, **P < 0.01, and ***P < 0.001 by 2-tailed *t* test or 1-way ANOVA. The test in panel **B** is 1-way ANOVA and in panels **C** and **D** is Mann-Whitney *U* test. For panel **E**, scale bar is 200 μm; cartilage and synovial tissues from *n* = 5 individuals were analyzed; and the representative images were shown.

To gain insight into the role of fibrinolysis in OA, we investigated whether genes involved in the fibrinolysis pathway were dysregulated in OA joints. Unsupervised hierarchical clustering analysis of gene expression microarray data sets on synovial membranes from individuals with early- or end-stage OA and healthy individuals revealed an upregulation of uPA and uPAR in the early- or end-stage OA joints (Figure 1A). No mRNA expression of plasminogen was detected in the synovial tissues from knee joints of either OA or other joint diseases, suggesting that plasminogen translocates into the synovium from circulation. Bulk RNA-sequencing data comparing gene expression in degenerative meniscus tear (DMT), rheumatoid arthritis (RA), and OA showed that *PLAU* expression was significantly upregulated in OA compared with DMT, with minor or no changes in *PLAU* receptor (*PLAUR*) (Supplemental Figure 1; supplemental material available online with this article; https://doi.org/10.1172/jci.insight.173603DS1).

Next, we determined if fibrinolysis protein levels were also dysregulated in OA joints. Higher levels of plasmin were detected in the synovial fluids (SFs) of individuals with OA when compared with healthy individuals by ELISA analysis. Plasmin levels were elevated in the SFs of individuals with anterior cruciate ligament (ACL) tear, who are at high risk for OA, and those with DMT, reflective of early OA, as compared with healthy individuals (Figure 1B). Plasmin levels from SFs of patients with ACL tear and DMT were significantly lower compared with OA. Our results showed that there was an increase in plasmin levels following traumatic joint injury (ACL), during early-stage OA (DMT), and during end-stage OA (Figure 1B). We also found higher levels of uPA and uPAR in the SFs of individuals with OA, when compared with plasma (Figure 1, C and D).

Corroborating these results, immunohistochemical analysis of damaged cartilage and synovium from individuals with OA showed positive staining of tPA, uPA, and uPAR compared with isotype controls (Figure 1E). Our data indicate that fibrinolysis is aberrant in OA joints.

### Genetic deficiency or pharmacological blockade of plasmin attenuates OA in mice.

**Figure 2:**
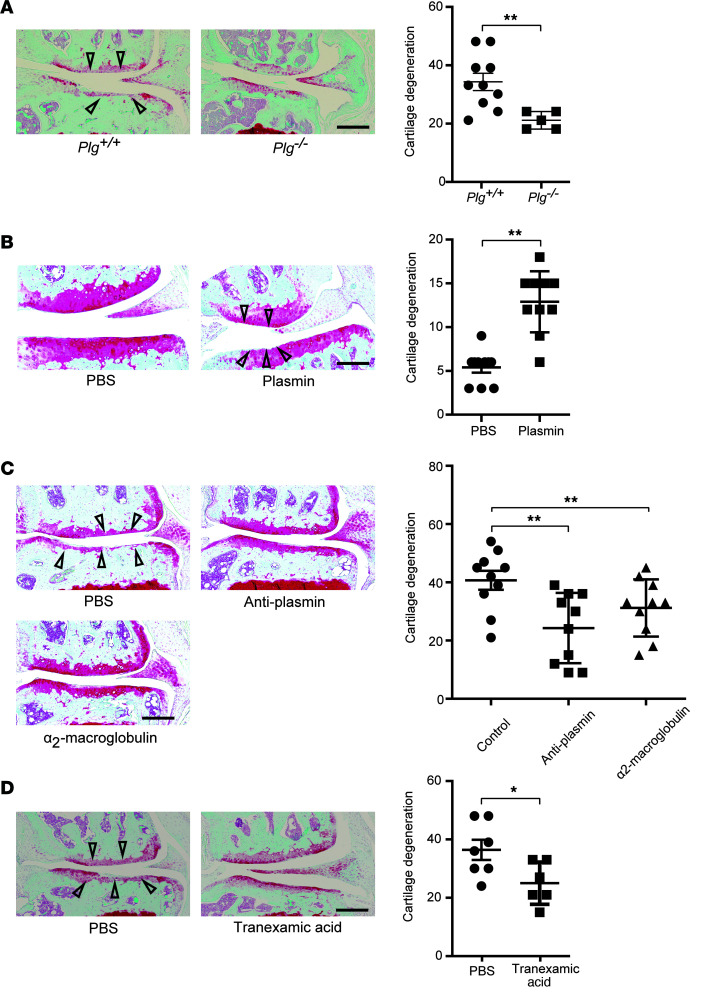
Genetic deficiency or pharmacological blockade of plasmin attenuates OA in mice. (**A**) Representative cartilage degeneration in Safranin-O–stained sections of the medial region of stifle joints from *Plg^+/+^* (*n* = 10) and *Plg^–/–^* (*n* = 5) male mice 20 weeks after DMM and quantification of the cartilage degeneration. (**B**) Representative cartilage degeneration in Safranin-O–stained sections of the medial region of stifle joints from C57BL/6J mice treated for 12 weeks with intra-articular injected plasmin (*n* = 10) or PBS (*n* = 10) and quantification of the cartilage degeneration. (**C** and **D**) Representative cartilage degeneration in Safranin-O–stained sections of the medial region of stifle joints from C57BL/6J mice subjected to DMM and treated with intra-articular injection of antiplasmin (*n* = 10), α2-macroglobulin (*n* = 10), or PBS (*n* = 10) (**C**) or with i.p. injection of tranexamic acid (*n* = 6) or PBS (*n* = 6) (**D**) for 12 weeks and quantification of the cartilage degeneration. Arrowheads indicate areas of cartilage degeneration. Scale bars, 200 μm. All data are the mean ± SEM of triplicates and are representative of 3 independent experiments. *P < 0.05, **P < 0.01. The test in panels **A**, **B**, and **D** is Mann-Whitney *U* test. Panel **C** uses 1-way ANOVA.

To understand the role of plasminogen and plasmin in the pathogenesis of posttraumatic OA, we examined the effects of genetic deficiency in the plasminogen gene *Plg* on the development of OA in the DMM mouse model. We surgically induced OA through DMM in both B6.129P2-*Plg^tm1Jld^*/J (*Plg^–/–^*) and *Plg^+/+^* mice. Twenty weeks after DMM, *Plg^–/–^* mice showed significantly less cartilage degeneration than *Plg^+/+^* mice (Figure 2A). Consistent with the protection seen in *Plg^–/–^* mice, intra-articular injection of plasmin for 12 weeks alone was sufficient to promote cartilage degeneration in C57BL/6J mice (Figure 2B). Additionally, intra-articular treatment with antiplasmin or α2-macroglobulin, a plasmin inhibitor (30), for 12 weeks following DMM, attenuated the progression of OA in C57BL/6J mice (Figure 2C). Furthermore, treatment with tranexamic acid, an antifibrinolytic (31), for 12 weeks mitigated cartilage degeneration in C57BL/6J DMM mice (Figure 2D). Taken together, these results show that plasmin plays a central role in the pathogenesis of OA.

### Genetic deficiency of PAI-1 accelerates OA while deficiency of tPA attenuates OA in DMM mice.

**Figure 3:**
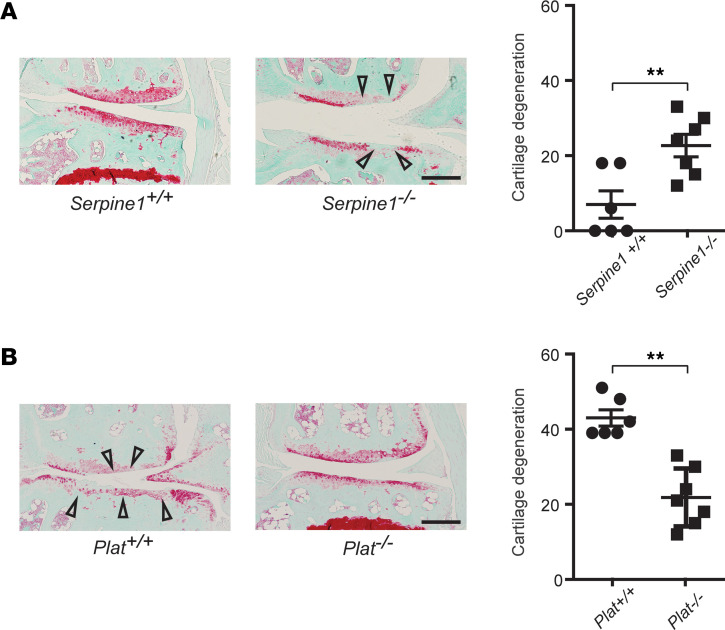
Genetic deficiency of PAI-1 accelerates OA while deficiency of tPA attenuates OA in DMM mice. (**A** and **B**) Representative cartilage degeneration in Safranin-O–stained sections of the medial region of stifle joints from *Serpine1^+/+^* (*n* = 7) and *Serpine1^–/–^* (*n* = 8) mice (**A**) or *Plat^+/+^* (*n* = 6) and *Plat^–/–^* (*n* = 7) mice (**B**) 20 weeks after DMM and quantification of the cartilage degeneration. Arrowheads indicate areas of cartilage degeneration. Scale bar, 200 μm. All data are the mean ± SEM of duplicates or triplicates and are representative of at least 2 independent experiments. **P < 0.01 by Mann-Whitney *U* test.

Next, we determined whether regulators of plasmin activation contribute to OA development. We tested whether genetic deficiency in either the plasmin inhibitor, PAI-1, or in the tissue plasminogen activator, tPA, affected the development of OA in a DMM mouse model. Mice that were genetically deficient for PAI-1 (B6.129S2-*Serpine1^tm1Mlg^*/J; *Serpine1^–/–^*) displayed more cartilage degeneration when compared with their wild-type (*Serpine1^+/+^*) littermates, 20 weeks after DMM (Figure 3A). In contrast, mice deficient in tPA (C.129S2-*Plat^tm1Mlg^*/J; *Plat^–/–^*) had less cartilage degeneration than their wild-type (*Plat^+/+^*) littermates when subjected to DMM (Figure 3B). Our data further indicate that the dysregulation of plasmin activation functions in the development of OA.

### Plasmin contributes to the initiation and progression of OA through multiple mechanisms: the degradation of lubricin and cartilage proteoglycan, activation of pro-MMPs, and induction of inflammatory and degradative mediators.

**Figure 4:**
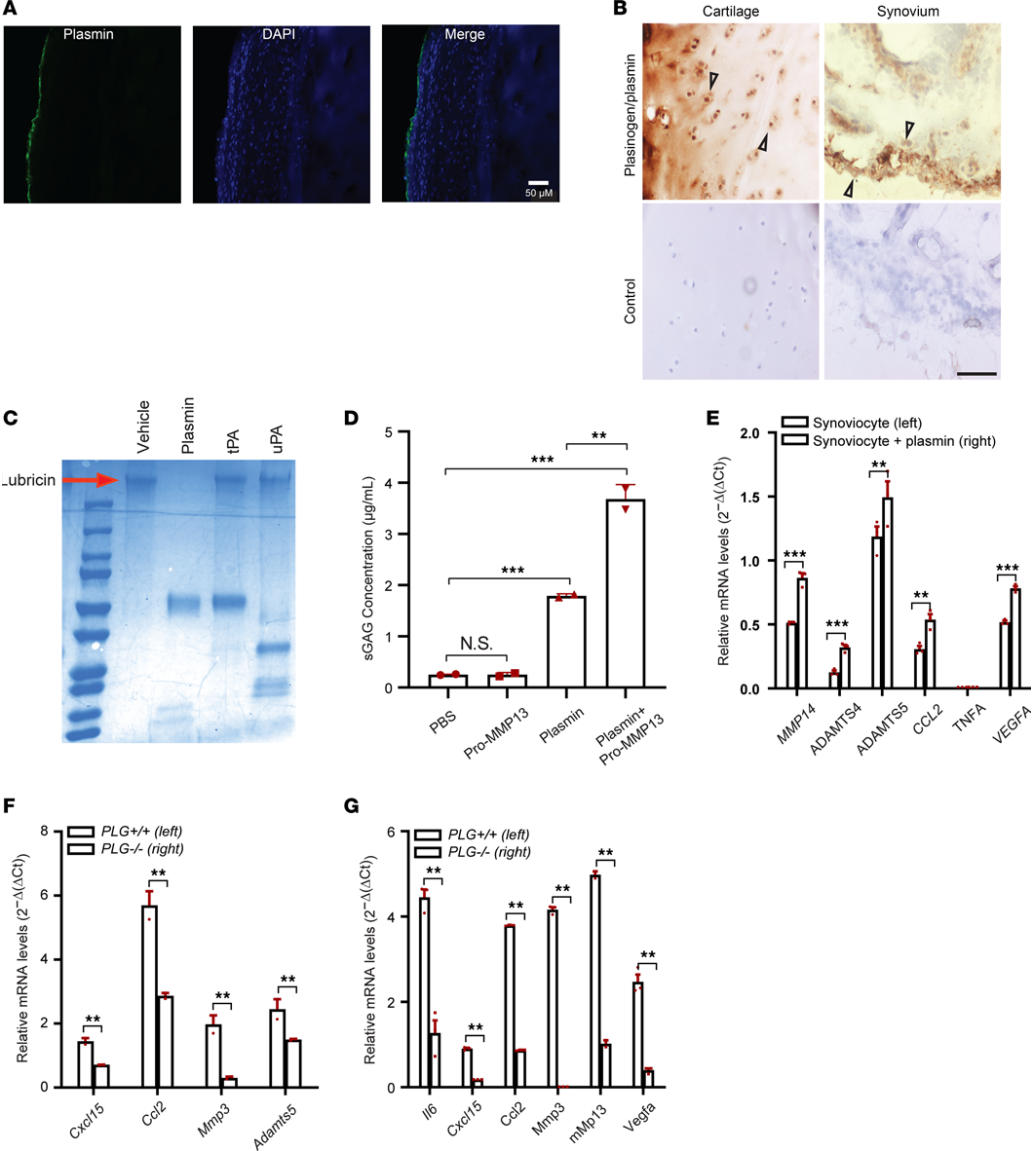
Plasmin contributes to the initiation and progression of OA through multiple mechanisms: the degradation of lubricin and cartilage proteoglycan, activation of pro-MMPs, and induction of inflammatory and degradative mediators from synovial cells. (**A**) Representative images from immunofluorescence staining of plasmin (green, left), staining of nuclei (blue, middle), and merging (right) in the undamaged articular cartilage area from individuals with knee OA who underwent total knee replacement. (**B**) Representative images from immunohistochemical staining of plasmin in the damaged articular cartilage area (upper left), the synovium (upper right), and the isotype controls (bottom, respectively) from individuals with OA. The arrowhead indicates binding of plasmin on the surface of chondrocytes (upper left) and cells of the synovial lining (upper right). (**A** and **B**) Scale bar, 200 μm; cartilage and synovial tissues from *n* = 5 individuals were analyzed. (**C**) Degradation of recombinant lubricin, shown on SDS-PAGE gel stained with Coomassie blue, by plasmin, but not activated tPA or uPA after 4 hours’ 37°C incubation. Red arrowhead shows the lubricin stained with Coomassie blue in different conditions: vehicle, plasmin, tPA, and uPA. (**D**) ELISA quantification of soluble sGAG released from cartilage explants from individuals with OA, treated with vehicle, pro–MMP-13, plasmin, or plasmin + pro–MMP-13. (**E**) Quantitative PCR (qPCR) analysis of OA-related inflammatory and degradative mediators as well as VEGFα in human primary synoviocytes, derived from the knee joints of individuals with OA, with or without plasmin stimulation. (**F** and **G**) qPCR analysis of relative gene expression levels of OA-related inflammatory and degradative mediators in synovial tissue (**F**) or articular cartilage from *Plg^+/+^* (*n* = 5) and *Plg^–/–^* (*n* = 5) mice 20 weeks after DMM. All data are the mean ± SEM of triplicates and are representative of 3 independent experiments. **P < 0.01, and ***P < 0.001. The test in panel **D** is 1-way ANOVA. The test in panels **E**–**G** is 2-tailed *t* test.

Given that aberrant activation of plasmin played a role in the pathogenesis of OA, we investigated how OA development was affected. We used immunohistochemistry to visualize the localization of plasminogen/plasmin in articular cartilage from individuals with OA. We found that plasminogen/plasmin was localized on the surfaces of cartilage in undamaged areas, while plasminogen/plasmin was undetected on chondrocytes located inside this area (Figure 4A). In contrast, we observed positive staining of plasminogen/plasmin on chondrocytes within the damaged cartilage area and synovium of individuals with OA (Figure 4B). These findings suggest that the superficial articular cartilage is resistant to damage caused by plasmin, and once the superficial cartilage is degraded, plasmin will continue to damage chondrocytes. Plasmin on synovial cells influenced synovium hemostasis.

We then investigated potential mechanisms by which plasmin contributes to synovial hemostasis imbalance and cartilage degradation. Lubricin, produced by synovial cells and chondrocytes, is a surface-active mucinous glycoprotein that imparts boundary lubrication between joint surfaces. It plays a role in reducing friction and in maintaining a wear-resistant property in articulating joints (32). We tested plasmin’s ability to degrade recombinant lubricin in vitro. Upon incubation with plasmin, lubricin was undetected by Coomassie stain on an SDS-PAGE gel, whereas lubricin was present after incubation with PBS, activated tPA, and uPA (Figure 4C). This indicates that plasmin, but not activated tPA or uPA, degrades lubricin.

Sulfated glycosaminoglycans (sGAGs) are important molecular indicators of healthy cartilage and are present at lower concentrations in OA (33, 34). sGAG, released from the cartilage by degradation, is an indicator of proteoglycan damage. We found that treatment with plasmin of cartilage explants from individuals with OA resulted in increased sGAG release when compared with cartilage treated with PBS (Figure 4D). Additionally, proteinases, such as MMPs (e.g., MMP-13) produced by chondrocytes and synovial cells in the joint, play a significant role in the pathological destruction and eventual loss of cartilage in OA (35). Incubation of cartilage explants with zymogen pro–MMP-13 alone did not lead to a higher release of sGAG. However, coincubation of plasmin and pro–MMP-13 led to a marked increase in sGAG release when compared with plasmin alone (Figure 4D). Further, no changes in sGAG levels were detected when cartilage explants were treated with activated tPA, in either the presence or absence of pro–MMP-13 (Supplemental Figure 2). These results are consistent with the notion that plasmin can degrade proteoglycans and other connective tissue components (36) and that plasmin can activate the latent form of MMPs, which can specifically degrade the ECM (37, 38).

In addition to the effect of plasmin on lubricin degradation and cartilage proteoglycan degradation, we examined whether it could directly induce production of inflammatory and degradative mediators in the joint. When stimulated with plasmin, human primary synoviocytes derived from the knee joints of individuals with OA expressed OA-related inflammatory *CCL2* and degradative *MMP14*, *ADAMTS4*, and *ADAMTS5* mediators as well as *VEGFA* (Figure 4E). Expression levels of inflammatory *Cxcl15* and *Ccl2* and degradative *Mmp3* and *Adamts5* mediators were significantly reduced in synovial tissue (Figure 4F) and *Il6*, *Cxcl15*, *Ccl2*, *Mmp3*, *Mmp13*, and *Vegf*α in articular cartilage (Figure 4G) from *Plg^–/–^* mice as compared with *Plg^+/+^* control mice 20 weeks after DMM. Together, our results indicate that plasmin promotes inflammatory and degradative mediators in OA joints, which may contribute to the pathogenesis of OA.

### Fibrinolysis molecule, uPA, and its receptor, uPAR, also play a critical role in the pathogenesis of OA.

**Figure 5:**
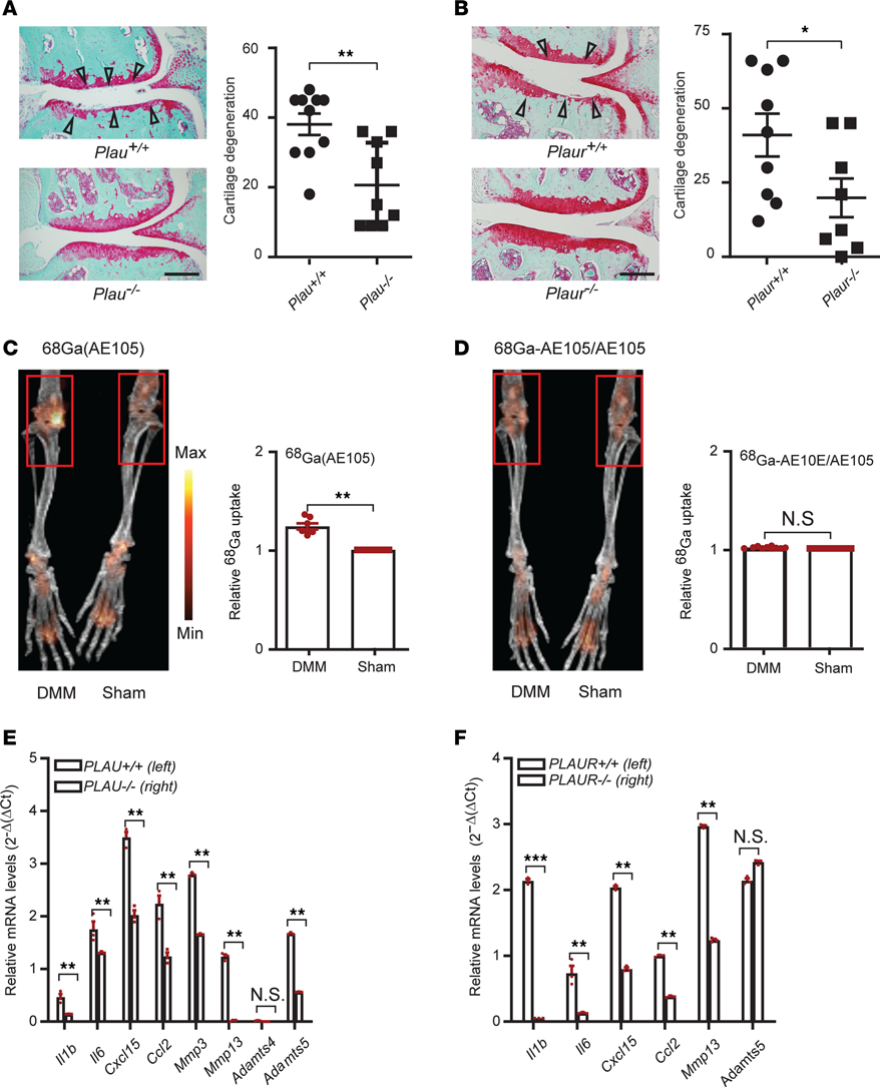
Another key fibrinolysis molecule, uPA, and its receptor, uPAR, also play critical roles in the pathogenesis of OA. (**A** and **B**) Representative cartilage degeneration in Safranin-O–stained sections of the medial region of stifle joints from *Plau^+/+^* (*n* = 10) and *Plau^–/–^* (*n* = 9) (**A**) and *Plaur^+/+^* (*n* = 9) and *Plaur^–/–^* (*n* = 9) mice (**B**) 20 weeks after DMM and quantification of the cartilage degeneration. Arrowheads indicate areas of cartilage degeneration. Scale bar, 200 μm. (**C** and **D**) MicroPET/CT imaging of mouse knee joints 20 weeks after DMM or sham surgery and quantification of relative ^68^Ga uptake levels in these joints (*n* = 7). Mice were i.v. injected with ^68^Ga-NODAGA-AE105 (**C**) or ^68^Ga-NODAGA-AE105 plus unlabeled AE105 (**D**). (**E** and **F**) qPCR analysis of relative mRNA expression levels of OA-related inflammatory, degradative mediators as well as VEGFα in synovial tissues from *Plau^+/+^* (*n* = 5) and *Plau^–/–^* (*n* = 5) mice (**E**) or *Plaur^+/+^* (*n* = 5) and *Plaur^–/–^* (*n* = 5) mice (**F**) 20 weeks after DMM. Data are the mean ± SEM of duplicates or triplicates and are representative of at least 2 independent experiments. *P < 0.05, ** *P* < 0.01, and *** *P* < 0.001. The test in panels **A** and **B** is Mann-Whitney *U* test. The test in panels **C**–**F** is 2-tailed *t* test.

After detecting elevated levels of uPA and uPAR in OA joints, we determined if genetic loss of function impacted the progression of OA in mice following DMM. We found that genetic deficiency of uPA (B6.129S2-*Plau^tm1Mlg^*/J; *Plau^–/–^*) and uPAR (B6.129P2-*Plaur^tm1Jld^*/J; *Plaur^–/–^*) decreased cartilage degeneration as compared with their wild-type littermates, 20 weeks after DMM (Figure 5, A and B), suggesting the involvement of both uPA and uPAR in the pathogenesis of OA.

Next, we studied uPA and uPAR binding in OA joints by quantifying the uptake of AE105, a core uPA/uPAR binding peptide, in the articular joints of C57BL/6J DMM mice. Radiolabeled AE105, ^68^Ga-NODAGA-AE105 (39), was injected i.v. 20 weeks after DMM or sham surgery. Deposition of AE105 was determined by microPET/CT imaging of mouse knee joints. Mice subjected to DMM took up a higher concentration of ^68^Ga-NODAGA-AE105 compared with the sham-operated side of joints (Figure 5C). Coinjection of competing, nonradioactive labeled AE105 diminished the difference in radiolabeled uPA uptake, verifying the binding specificity of AE105 to uPAR (Figure 5D). Our data show that uPA/uPAR binding increased in a mouse model of DMM-induced OA.

We also examined if genetic deficiencies in uPA and uPAR mitigated DMM-induced OA pathologies by affecting the production of inflammatory and degradative mediators by cells in the joint. Indeed, reduced gene expression levels of OA-related inflammatory *I1b*, *Il6*, *Cxcl15*, and *Ccl2* and degradative *Mmp3*, *Mmp13*, and *Adamts5* mediators, but not *Adamts4*, were detected in synovial tissues from *Plau^–/–^* DMM mice (Figure 5E). Reduced gene expression levels of OA-related inflammatory *Il1b*, *Il6*, *Cxcl15*, and *Ccl2* and degradative *Mmp13* and *Adamts5* mediators were also detected in synovial tissues from *Plaur^–/–^* mouse knee joints subjected to DMM (Figure 5F).

### uPA induces inflammatory and degradative mediators via PI3K and AKT downstream signaling pathways to promote OA.

**Figure 6:**
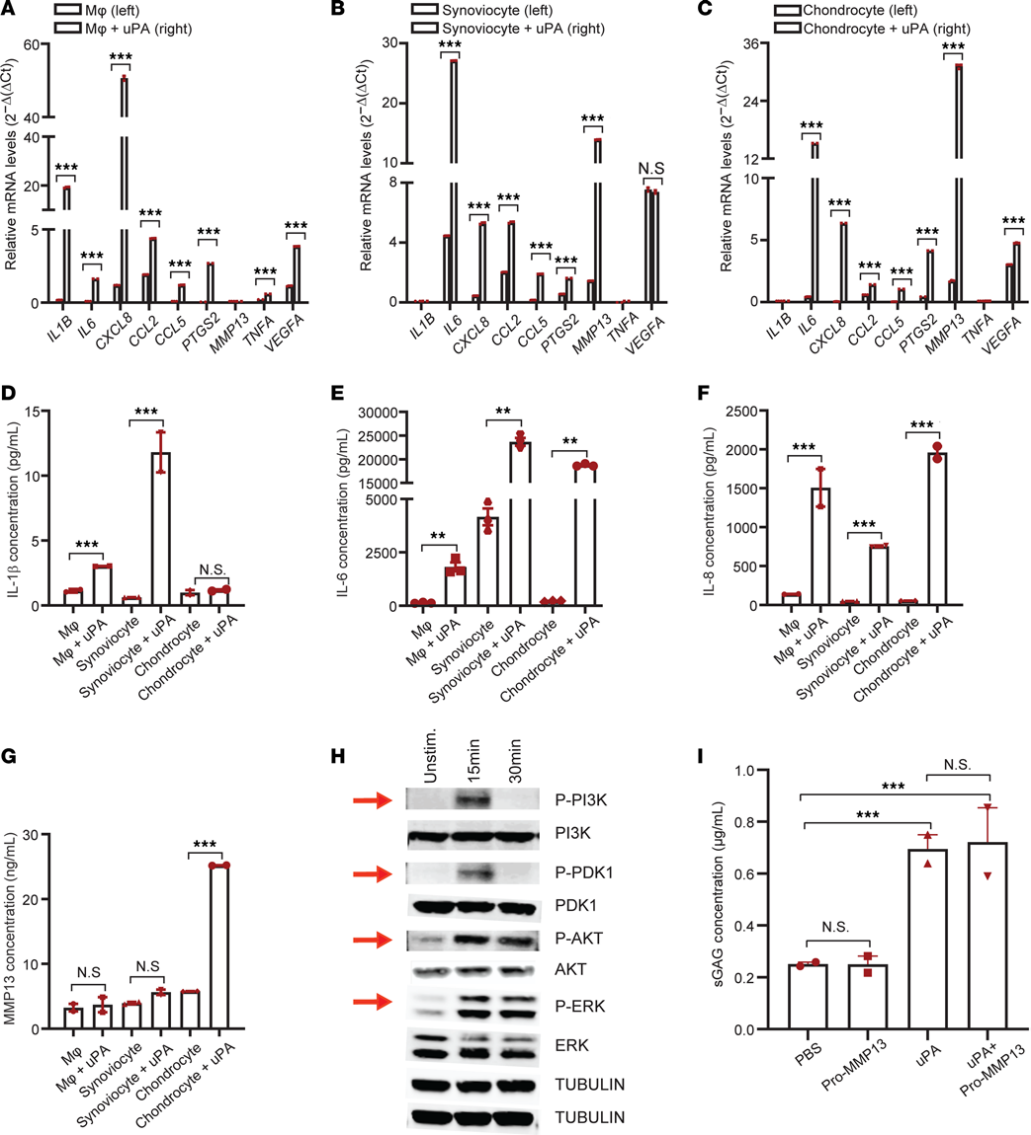
uPA induces inflammatory and degradative mediators via PI3K and AKT downstream signaling pathways to promote OA. (**A**–**C**) qPCR analysis of OA-related inflammatory and degradative mediators as well as VEGFα in human monocyte-derived macrophages (**A**), primary synoviocytes (**B**), and cartilage chondrocytes (**C**); all cells except macrophages are derived from the knee joints of individuals with OA who underwent total knee replacement, stimulated with or without uPA. (**D**–**H**) ELISA validation of protein levels of IL-1β (**D**), IL-6 (**E**), IL-8 (**F**), and MMP-13 (**G**) in human macrophages stimulated with or without uPA. All data are the mean ± SEM of triplicates and are representative of 3 independent experiments. (**H**) Western blot analysis of phosphorylation of signaling molecules in human primary synoviocytes, derived from the knee joints of individuals with OA, that were either unstimulated or stimulated with uPA (15 minutes and 30 minutes). Red arrows denote changes in phosphorylation levels over time. Data are representative of at least 3 independent experiments. (**I**) ELISA quantification of soluble sGAG released from cartilage explants from individuals with OA, treated with PBS, pro–MMP-13 alone, uPA alone, or uPA and pro–MMP-13 together. All data are the mean ± SEM of triplicates and are representative of 3 independent experiments. **P < 0.01, and ***P < 0.001. The test in panels **A**–**G** is 2-tailed *t* test. The test in panel **I** is 1-way ANOVA.

After finding that mouse synovial tissue deficient for uPA and uPAR had reduced expression of inflammatory and degradative mediators, we tested whether uPA was able to induce production of these mediators in different cell types within the joint. We observed an increase in expression of *IL1B*, *IL6*, *CXCL8* *CCL2*, *CCL5*, *PTGS2*, and *VEGFA* when human monocyte-derived macrophages were stimulated with uPA (Figure 6A). A similar transcriptomic signature was detected in synoviocytes with a marked upregulation of *ILB*, *IL6*, *CXCL8*, *CCL2*, *CCL5*, *PTGS2*, and *MMP13* but not *TNFA* or *VEGFA* (Figure 6B). In cartilage chondrocytes from OA knee joints, we noted a significant increase in *IL6*, *CXCL8*, *CCL2*, *CCL5*, *PTGS2*, *MMP13*, and *VEGFA* (Figure 6C). In some cases, elevated transcription led to an increase in protein translation as measured by ELISA (Figure 6, D–G). Higher IL-1β protein concentration was detected in uPA-stimulated chondrocytes, while higher IL-6 and IL-8 were seen in uPA-stimulated macrophages, synoviocytes, and chondrocytes, as well as increased pro–MMP-13 protein levels in uPA-stimulated synoviocytes. The induction of inflammatory and degradative mediators by uPA is similar to the effect of plasmin that we observed before (Figure 4E).

In light of the above findings, we investigated the intracellular pathways through which uPA induces changes in the expression of inflammatory and degradative genes in OA. Upon stimulation with uPA, human primary synoviocytes, derived from the knee joint of individuals with OA, showed transient increases in PI3K phosphorylation and PDK1, 15 minutes after stimulation. AKT and ERK remained phosphorylated at 15 and 30 minutes after stimulation (Figure 6H). Our data suggest that, analogous to the mechanism in other diseases such as cancer (40), uPA activates a cascade of known target molecules and thereby induces inflammation and tissue degradation in the OA joint.

Finally, we also investigated the role of uPA in cartilage degradation in OA (Figure 6I). We found that treatment of cartilage explants from individuals with OA with uPA resulted in increased release of sGAG into the supernatant of cultures, while incubation of cartilage explants with pro–MMP-13 and uPA did not lead to an increase in sGAG release when compared with cartilage treated with PBS and pro–MMP-13 (Figure 4D). Our data suggest that similar to plasmin, uPA can directly degrade certain proteoglycans (41), despite its inability to degrade lubricin (Figure 4C), and cannot activate MMP zymogens to further degrade proteoglycan.

## Discussion

No effective treatment is currently approved to prevent or to slow progression of OA. This is largely due to an incomplete understanding of the underlying mechanisms of OA pathogenesis and disease progression. Here, we showed through transcriptomic and immunohistochemical analyses of human OA joints, as well as analysis of the effects of genetic deficiencies and pharmacological treatment of mice following DMM, that the fibrinolytic pathway, which includes plasmin, uPA, and uPAR, contributes to the pathogenesis of OA after joint injury. Our in vitro studies revealed that plasmin is: i) a powerful catabolic enzyme that degrades lubricin and cartilage proteoglycans, ii) a potent activator of pro-MMPs, and iii) a robust inducer of inflammatory and degradative mediators. We also showed that uPA can degrade cartilage proteoglycans, activate pro-MMPs, and induce expression of inflammatory and degradative mediators through downstream PI3K signaling pathways. These findings provide rationale for targeting fibrinolytic molecules and pathways as disease-modifying therapies to prevent or treat posttraumatic OA.

It was previously shown that loss of function of PAI-1 accelerated subchondral osteopenia in mice, suggesting that PAI-1 suppresses bone resorption after OA induction (42). Another group reported that uPAR expression was significantly higher in fibroblast-like synoviocytes (FLSs) in patients with RA, compared with FLSs in OA. uPAR plays a seemingly important role in FLSs in RA and may be associated with the β1-integrin/PI3K/AKT signaling pathway (43). Our work indicates that plasmin, uPA, and uPAR contribute to the pathogenesis of OA.

Multiple antifibrinolytics are currently available as FDA-approved or investigational drugs (44, 45). Given the role of fibrinolysis in posttraumatic OA, novel antifibrinolytic therapeutics administered after joint injury may offer new opportunities to prevent the onset of or halt the progression of OA. Strategies that use these antifibrinolytic drugs could form the basis of a much-needed disease-modifying therapy for posttraumatic OA (46). Tranexamic acid is a synthetic analog of the amino acid lysine. It serves as an antifibrinolytic by reversibly binding 4 to 5 lysine receptor sites on plasminogen. This decreases the conversion of plasminogen to plasmin. In this study, we showed that tranexamic acid can slow development of posttraumatic OA in a DMM mouse model. The effectiveness of tranexamic acid as a treatment for posttraumatic OA after ACL and/or DMT tear is currently being tested in a clinical trial (ClinicalTrials.gov Identifier: NCT03552705).

Here, we identified plasmin as a key regulator of OA development following joint injury. Aberrant plasmin activity was detected in the OA joint (29, 47), but until this report, the function of activated plasmin in OA pathogenesis remained unclear. Previous reports described a role for plasmin in degrading ECM components of cartilage and lubricin (36, 48, 49) and in activating MMPs (37, 50–52). Guided by these observations, we found that plasmin is pathogenic in the development of posttraumatic OA and that it promotes disease progression through several mechanisms. Immediately after joint injury, plasmin activation at the site of injury ensures timely resolution of fibrin clot formation, which allows for wound healing and tissue restoration. Transient activation of plasmin is therefore protective. However, we showed that plasmin levels are elevated in the synovial fluids of those with ACL tears, DMTs, and OA joints, suggesting that continuous plasmin activation may be pathogenic. Additionally, we showed that plasmin also induces synovial inflammation (synovitis), a pivotal step in OA pathogenesis (53–56), by increasing the production of inflammatory and degradative mediators by cells in the joint. Our data indicated that plasmin accelerates cartilage degeneration by acting as a catabolic enzyme that directly degrades lubricin and proteoglycans (36). We also found that plasmin increases the production and activation of OA-related degradative enzymes (MMPs), which in turn causes further damage to the cartilage (37, 38, 57). Overall, we demonstrated the multifaceted role of plasmin as both an initiator and propagator of cartilage destruction and joint inflammation in posttraumatic OA.

Our analysis on human OA synovial tissues, SFs, mouse genetics, and ex vivo microPET/CT showed that uPA is a key factor in the progression of joint injury to OA. We found that similar to plasmin, the contribution of uPA to the pathogenesis of OA promotes both cartilage degradation and inflammation. Data from this study showed that uPA directly degrades proteoglycans (41). Additionally, we showed that uPA also served as a zymogen activator for pro-MMPs. Finally, by interacting with its receptor, uPAR, uPA triggered the activation of downstream signaling molecules, including PI3K, PDK1, AKT, and ERK, to induce the production of inflammatory and degradative enzymes by cells in the joint.

**Figure 7:**
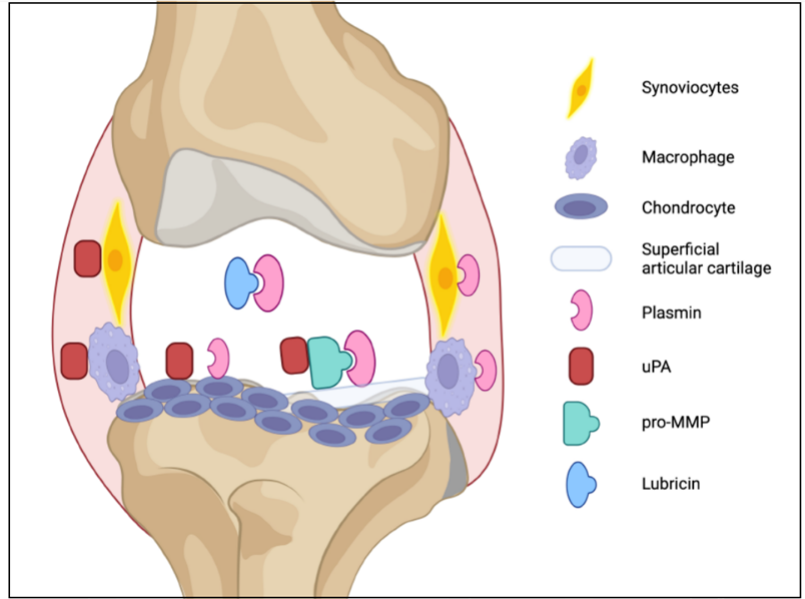
Targeted therapy for knee articular cartilage homeostasis. Overview of plasminogen effectors, enzymes, and proteins that can be targeted by pharmacological inhibitors to restore cartilage homeostasis and treat osteoarthritis.

Treatment of OA has not changed much over the last couple of years. Options for OA remain limited to pain management with analgesic drugs and mitigation of inflammation with glucocorticoids. Localized, intra-articular injections have major advantages over systemic drug administration. Treatment of OA by targeted therapy provides a promising alternative to traditional shotgun approaches. A healthy joint comprises a balance between cartilage production and cartilage breakdown. Inhibitors of fibrinolysis pathway molecules will restore articular chondrocyte homeostasis, encourage deposit of lubricin and sGAG in the ECM, and lessen the effects of OA in traumatic joints. In this study, we focused on localized, intra-articular administration of several potential drugs or molecules, such as tranexamic acid and α2-macroglobulin. Therapeutics that target specific molecules like uPA or uPAR can be used in combination with antiplasmin as a targeted therapy for OA (Figure 7).

Together, our results demonstrate that the fibrinolysis pathway, which includes plasmin, uPA, and uPAR, plays an important role in posttraumatic OA. Targeted therapy against these molecules may slow down OA disease progression after joint injury.

## Methods

### Human samples.

SF, synovium, and cartilage samples were obtained from individuals with knee OA who underwent total knee arthroplasty or surgeries to treat ACL injury and DMT. The patients were recruited from Palo Alto VA Medical Center. Human PBMCs were obtained from the Stanford Blood Bank.

### Transcriptional profiling.

We analyzed mRNA from osteoarthritic synovial membranes using Affymetrix Human U133 Plus 2.0 arrays (NCBI Gene Expression Omnibus [GEO] GSE32317). Data from healthy synovial membranes (analyzed on the same platform and array) were downloaded from the NCBI (GEO GSE12021) (58). We merged these data sets (59), computed the robust multichip average expression of genes, and organized transcript expression profiles by unsupervised or supervised hierarchical clustering (Cluster 3.0, http://bonsai.hgc.jp/~mdehoon/software/cluster/software.htm).

### ELISA.

The concentrations of plasmin (Anaspec), uPA, uPAR (R&D Systems), IL-1β, IL-6, IL-8 (PeproTech), and MMP-13 (Anaspec) were determined by ELISA according to the manufacturers’ instructions.

### QPCR.

RNA was isolated from cultured cells using RNeasy Plus Mini Kit (QIAGEN); reverse transcription was performed using oligo(dT)_18_ (Applied Biosystems) and performed by qPCR (Applied Biosystems). Results were normalized to 18s or β-actin RNA: 2^−Δ(ΔCt)^. The following PCR primers were acquired commercially (Thermo Fisher Scientific) for use in this study: *Ilib* (catalog Mm00434228_m1), *IL1B* (catalog Hs01555410_m1), *Iil6* (catalog Mm00446190_m1), *IL6* (catalog Hs00985639_m1), *Ccl2* (catalog Mm00441242_m1), *CCL2* (catalog Hs00234140_m1), *Adamts4* (catalog Mm00556068_m1), *ADAMTS4* (catalog Hs00192708_m1), *Adamts5* (catalog Mm00478620_m1), *ADAMTS5* (catalog Hs01095518_m1), *Vegfa* (catalog Mm01281449_m1), *VEGFA* (catalog Hs00900055_m1), *Mmp13* (catalog Mm00439491_m1), *MMP13* (catalog Hs00233992_m1), *MMP14* (catalog Hs00237119_m1), *Ptgs2* (catalog Mm00478374_m1), *PTGS2* (catalog Hs00153133_m1), *Cxcl15* (catalog Mm00441263_m1), *CXCL8* (catalog Hs00174103_m1), *CCL5* (catalog Hs00982282_m1), *Tnfa* (catalog Mm00443258_m1), *TNFA* (catalog Hs00174128_m1), *Actb* (catalog Mm00607939_s1), and *18S* (catalog Hs99999901_s1).

### Immunohistochemistry.

Human OA synovial tissue or frozen cartilage sections were fixed in 4% paraformaldehyde, stained with mouse anti-human plasmin monoclonal antibody (1:200) (Abcam ab10178), rabbit anti-human uPA monoclonal antibody (1:100) (Abcam ab133563), mouse IgG1 isotype control (1:100) (DAKO X093101-2), and rabbit IgG isotype control (1:1,000) (Abcam ab99234), followed by staining with secondary antibodies goat anti-mouse IgG (1:1,000; Abcam ab97040) conjugated with either Alexa Fluor 488 or HRP (Life Technologies A-11029 and Abcam ab97080, respectively). Cell nuclei were counterstained with DAPI (Life Technologies) or hematoxylin.

### Lubricin degradation experiment.

Human recombinant lubricin (20 μg) (gift from Lubris Biopharma, Weston, Massachusetts, USA) was incubated at 37°C with (i) recombinant human plasmin, (ii) tPA, and (iii) uPA for 4 hours. Supernatants were used to run SDS-PAGE gel, stained by Coomassie blue.

### SGAG content.

The content of sGAG was determined using Blyscan assay kits (Biocolor). Human OA knee articular cartilage was frozen and thawed 3 times, immersed in 95% ethanol for 3 hours at room temperature, and washed in PBS. Pieces of acellular cartilage of the same weight were incubated at 37°C for 4 hours in PBS or PBS with (i) recombinant human plasmin, (ii) uPA, (iii) pro–MMP-13, (iv) plasmin + pro–MMP-13, (iv) uPA + pro–MMP-13, (v) tPA, or (vi) tPA + pro–MMP-13. Supernatants were probed for sGAG content by the Blyscan assay kit according to the manufacturer’s instructions.

### Surgical mouse model of OA.

We generated the DMM model of OA according to established methods (60, 61), by using 20-week-old male mice. Experiments continued for 12 or 20 weeks after surgery. Mice deficient in plasminogen (*Plg^–/–^*) (B6.129P2-*Plg^tm1Jld^*/J), PAI-1 (*Serpine1*^–/–^) (B6.129S2-*Serpine1^tm1Mlg^*/J), tPA (*Plat^–/–^*) (C.129S2-*Plat^tm1Mlg^*/J), uPA (*Plau^–/–^*) (B6.129S2-*Plau^tm1Mlg^*/J), or uPAR (*Plaur^–/–^*) (B6.129P2-*Plaur^tm1Jld^*/J), as well as wild-type control (C57BL/6J) mice, were obtained from The Jackson Laboratory.

### Conjugation and radiolabeling of ^68^Ga-NODAGA-AE105.

High-affinity uPAR binding peptide AE105 (Elim Biopharm) was conjugated to the NODA-GA-NHS chelator (CheMatech) by classic click chemistry (62). Briefly, AE105 was dissolved in PBS (pH 7.4) at a concentration of 1 mg/mL. The bifunctional chelator NODA-GA-NHS ester was dissolved in DMSO (10 mM) and added to the peptide at a 20:1 molar ratio. Reagents were mixed by vortexing overnight at 4°C, then purified by HPLC. Characterization of the conjugated products was done by liquid chromatography–electrospray ionization–mass spectrometry. A total of 8 μg of NODAGA-AE105 was radiolabeled with ^68^Ga by the addition of 203.5–240.5 MBq (5.5–6.5 mCi) of ^68^GaCl_3_ in 1 M NaOAc (pH 4.5) buffer followed by a 15-minute incubation at 85°C (63). The radiolabeled complex was then purified by C-18 cartridge, dried by nitrogen, and reconstituted in PBS in sterile vials for future usage.

### MicroPET/CT.

Twenty weeks after DMM, microPET/CT imaging (Siemens Inveon microPET/CT) was performed using established methods (64). In one set of experiments, mice were injected with 7.4–14.8 MBq (200–400 μCi) ^68^Ga-labeled radiotracers (^68^Ga-NODAGA-AE105), in the absence or presence of the corresponding unlabeled blockers (100–300 μg per mouse), via the tail vein. In a second set of experiments, mice were injected with 7.4–14.8 MBq (200–400 μCi) ^68^Ga-NODAGA-AE105. Ten minutes after injection, the mice were sacrificed, legs were collected, and legs were placed near the center of the field of view of the scanner. All PET images were obtained through 5-minute static acquisition and reconstructed with IRW4.0 software by a 3-dimensional ordered-subset expectation maximization algorithm. The degree of radioactivity uptake in the knee joints was evaluated by analyzing the region of interest (ROI) drawn over the whole joint region; this ROI was the same for the DMM and sham-operated joints. The degree of radioactivity uptake in the DMM side was calculated as (radioactivity of DMM joint)/(radioactivity of sham-operated) × 100% for each mouse, with uptake in the sham-operated joint set at 100%.

### Scoring of cartilage degeneration in mouse OA.

We stained sections of mouse joints with Safranin-O. Cartilage degeneration was blindly scored by a trained examiner using a modified version of a previously described system (65): depth of cartilage degeneration (score of 0–4) × width of cartilage degeneration (with a score of 1 meaning one-third of the surface area, a score of 2 meaning two-thirds of the surface area, and a score of 3 meaning the whole surface area) in each third of the femoral-medial and tibial-medial condyles. The scores for the 6 regions were summed.

### Treatment of mouse OA.

We treated DMM C57BL/6J mice by injecting 7 μL twice/week intra-articularly with vehicle (PBS), 0.5 μg/mouse plasmin, 25 μg/mouse antiplasmin (Fitzgerald Industries International), or 1 μg/mouse α2-macroglobulin (Fitzgerald Industries International). A volume of 100 μL tranexamic acid (10 mg/kg) or vehicle (PBS) was administered i.p., twice a day for 12 weeks, starting 1 week after DMM.

### Isolation and culture of human primary chondrocytes.

Human primary chondrocytes were isolated from osteoarthritic cartilage as described (66). Briefly, OA articular cartilage was cut into small pieces, seeded in 10 cm Petri dishes in DMEM-F12 (Thermo Fisher Scientific) containing 10% FBS and 1 mg/mL collagenase II (Worthington Biochem), and incubated overnight at 37°C. Chondrocytes were cultured for 3–5 days, then seeded in 12-well plates, and adherent cells were treated with 0.5 U human recombinant plasmin (Abcam) or uPA (R&D Systems) for 24 hours for qPCR or ELISA analyses.

### Isolation, culture, and treatment of human primary synoviocytes.

Human primary synoviocytes were prepared as described (66). Briefly, synovial tissue was obtained from the osteoarthritic knee joint, cut into small pieces, and digested with 1 mg/mL collagenase IV (Worthington Biochem) in 10% FBS overnight at 37°C. Isolated primary synovial cells were cultured in DMEM with 10% FBS for 3–5 days. Adherent cells were treated in the absence or presence of 0.5 U human recombinant plasmin or uPA for 24 hours. Samples were collected at 0, 15 minutes, 30 minutes, and 24 hours and analyzed by qPCR, Western blot, or ELISA.

### Isolation, culture, and treatment of human macrophages.

PBMCs from healthy individuals were cultured with 25 ng/mL human recombinant M-CSF (PeproTech) for 14 days. Adherent cells were treated in the absence or presence of 0.5 U human recombinant or uPA for 24 hours, then analyzed by qPCR or ELISA. We used 1 × 10^6^ cells / mL for all macrophage experiments.

### Recombinant proteins used in this study.

We used recombinant human plasmin (Cell sciences, CRP135), tPA (Abcam, ab92633), uPA (BioLegend, 755302), pro–MMP-13 (MilliporeSigma CC1047), and plasmin (Abcam, AB84406).

### Western blots.

Western blot analysis was performed with anti–p-PI3K/anti-PI3K (no. 4228/no. 13666), anti–p-PDK1/anti-PDK1 (no. 3061/no. 3062), anti–p-AKT/anti-AKT (no. 9275/no. 2920), anti–p-ERK/anti-ERK (no. 9101/no. 4695) (Cell Signaling Technology), and Tubulin (Abcam ab59680).

### Statistics.

Single-comparison analyses were performed using a 2-tailed *t* test or Mann-Whitney *U* test following tests for variance homogeneity. One-way ANOVA was used for multiple comparisons followed by Dunnett’s post hoc test.

### Study approval.

This study and all protocols pertaining to our human and animal research were approved by Stanford University’s Institutional Review Board (IRB) and the Stanford Research Compliance Office. SF, synovial lining, cartilage, and PBMCs were collected with ethical approval from the Stanford University IRB. Written informed consent was received from all human participants prior to participation. We performed all mouse studies under protocols approved by the Stanford Committee of Animal Research and in accordance with the NIH *Guide for the Care and Use of Laboratory Animals* (National Academies Press, 2011) or the equivalent.

### Data availability.

Stanford Research Compliance Office provided guidance in the protection of the rights, welfare, and well-being of human participants and may choose not to make some patient or donor information available. Values for all data points found in graphs are in the Supporting Data Values file.

## Author contributions

QW and WHR designed the research project. QW, HHW, XZ, KC, MZ, and AB performed the research and analyzed the data. GS and ZC did the microPET study. QW, MSB, ZZL, and WHR wrote the manuscript. CRC provided samples and scientific guidance. All authors reviewed, provided edits and input on, and approved the manuscript.

## Supplementary Material

Supplemental data

Unedited blot and gel images

Supporting data values

## Version 1 03/19/2024

In-Press Preview

## Version 2 04/22/2024

Electronic publication
