# Supplementary material for: Dysregulated fibrinolysis and plasmin activation promote the pathogenesis of osteoarthritis
Source: JCI Insight. 2024 Mar 19;9(8):e173603. doi: 10.1172/jci.insight.173603 (PMC11141881; doi:10.1172/jci.insight.173603)
Supplement: Supplemental data [file jciinsight-9-173603-s174.pdf]

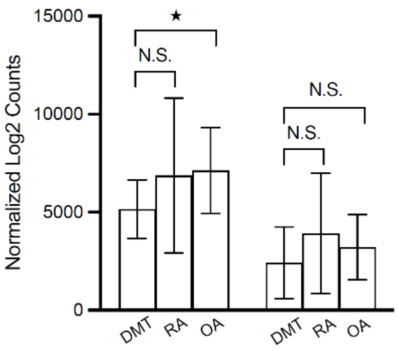

Supplemental Figure 1. **The gene expression of *PLAU* and *PLAUR from bulk RNA-seq*.**
The synovial tissue RNA was extracted from DMT, RA, and OA patients and sequenced. Data is presented as normalized log2 counts of *PLAU* and *PLAUR* expression in the synovium. Within the comparison, only the upregulated expression of *PLAU* in OA compared with DMT is significant. The rest are not. NS P > 0.05, *P ≤ 0.05, **P ≤ 0.01, and ***P ≤ 0.001 by one way ANOVA.

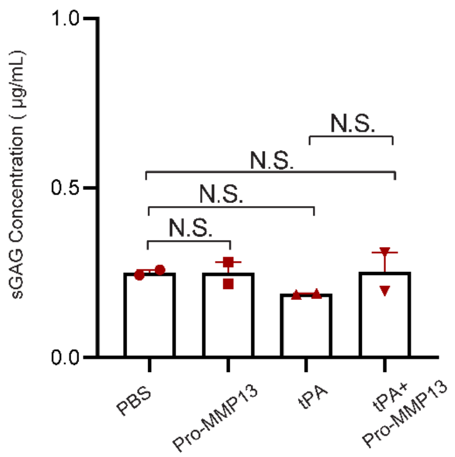

Supplemental Figure 2. Effects of tPA on the articular cartilage proteoglycan degradation.
ELISA quantification of soluble sGAG released from cartilage explants from individuals with OA, treated with PBS, pro-MMP13 alone, tPA alone, or tPA and pro-MMP13 together. All data are the mean ± SEM of triplicates and are representative of three independent experiments. NS $P > 0.05$, *$P \leq 0.05$, **$P \leq 0.01$, and ***$P \leq 0.001$ by one way ANOVA.
