# Supplementary figures and images for: Dysregulated fibrinolysis and plasmin activation promote the pathogenesis of osteoarthritis
Source: JCI Insight. 2024 Mar 19;9(8):e173603. doi: 10.1172/jci.insight.173603 (PMC11141881; doi:10.1172/jci.insight.173603)

P-AKT

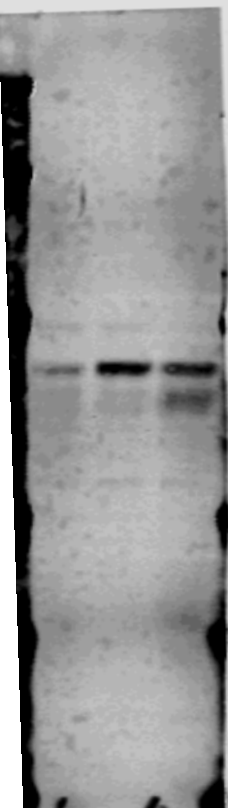

60KD

P-ERK

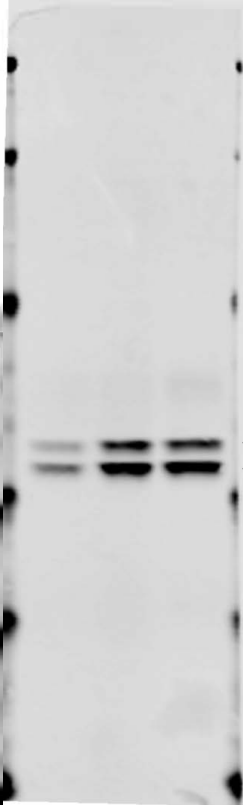

42KD

44KD

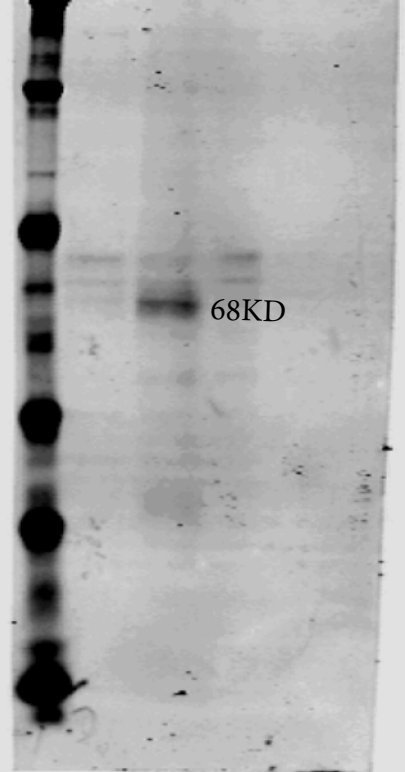

P-PDK1

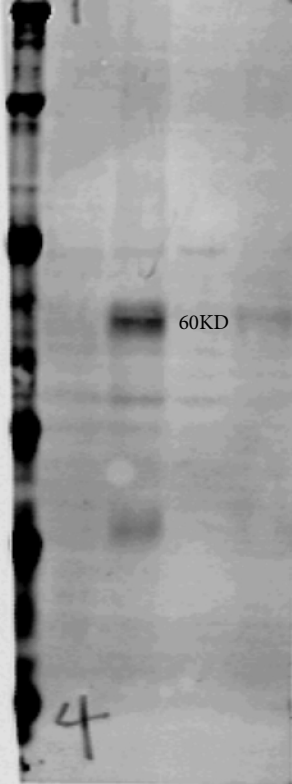

P-PI3K

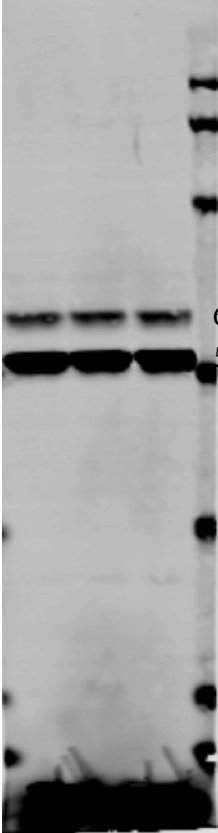

60 KD AKT

55KD Tubulin

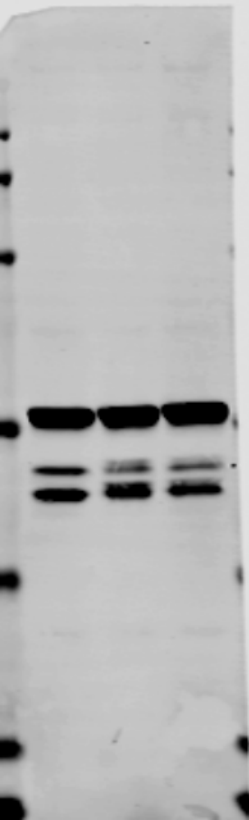

55KD Tubulin

42KD/44KD  
ERK

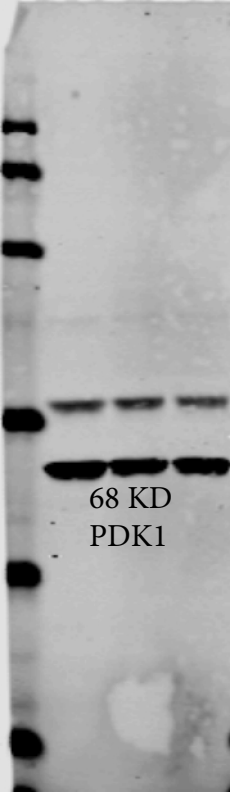

68 KD  
PDK1

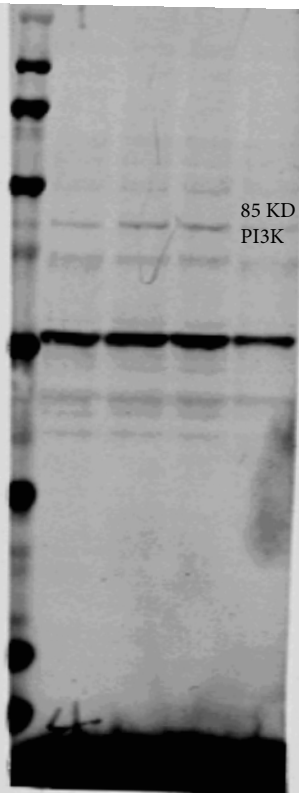

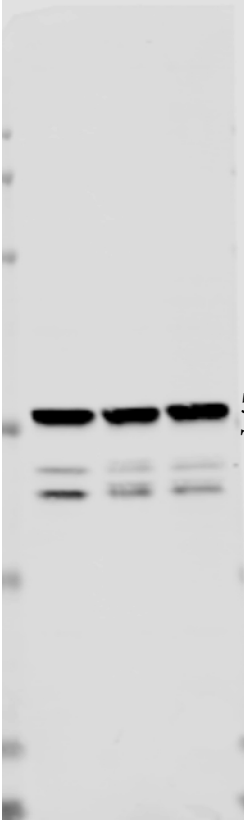

55KD  
Tubulin

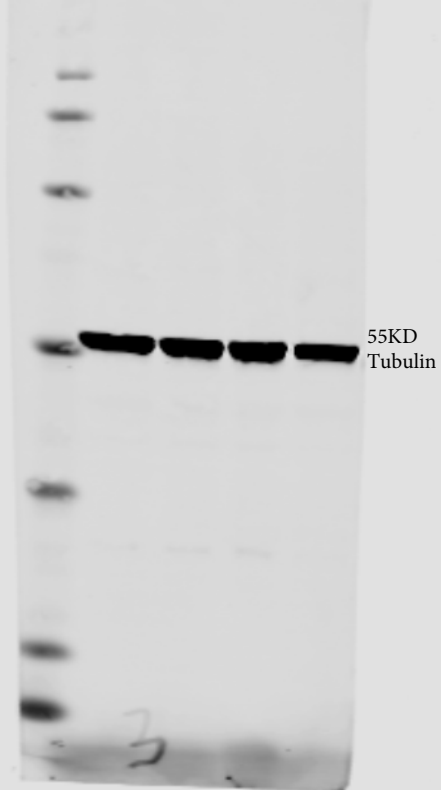

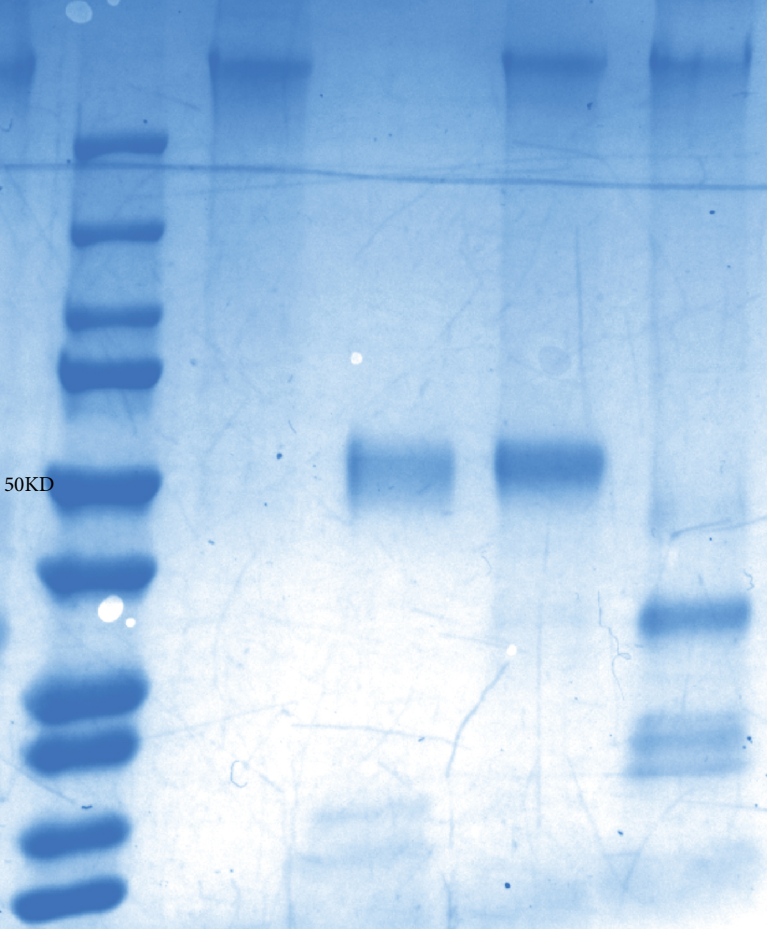

Supplement: Unedited blot and gel images [file jciinsight-9-173603-s175.pdf]
